# A case of a speech impediment following a near lightning strike

**DOI:** 10.1186/1865-1380-4-60

**Published:** 2011-09-19

**Authors:** Bobby K Desai, Rita Fairclough

**Affiliations:** 1 University of Florida College of Medicine Department of Emergency Medicine PO Box 100186 Gainesville, FL 32610, USA

## Abstract

Environmental electrical injuries (electrical burns and lightning) are relatively common and are estimated to result in more than 3,000 admissions to specialized burn units each year here in the US. Lightning injuries are a small subset of electrical injuries and are responsible for an average of 300 injuries and 100 deaths per year in the US. We present a case of a rare injury obtained as a result of a near lightning strike. The case involved a young female who was playing soccer when lightning struck within several feet of where she was standing, resulting in loss of consciousness, paresthesias, tinnitus, muscle spasms and most importantly a new onset of a speech impediment. There is only one reported case of a speech impediment secondary to an electrical injury in the literature.

## Introduction

Lightning injuries are responsible for an average of 300 injuries and 100 deaths per year [1]. Approximately 30 percent of patients that are struck die and up to 73 percent of patients that survive may have permanent disabilities [2]. Deaths can occur within 1 h of injury in the majority of cases, and are secondary to fatal arrhythmias or respiratory failure. Seventy-four percent of survivors experience permanent injury and sequelae as indicated by some reports [3].

More than one half of the fatalities occur while people are involved in outdoor activities, and another 25 percent occur during work-related activities.

Between the years of 1980 and 1996 the Centers for Disease Control reported 1,318 patients killed by lightning strikes, of which 85 percent were male. Within the United States, the areas with the greatest number of deaths secondary to lightning were Florida and Texas, and the states with the highest incidence of strikes were New Mexico, Arizona, Arkansas and Mississippi.

Injuries due to electricity occur by three mechanisms: Direct effect of current on body tissues; conversion of electrical energy to thermal injury resulting in burns; and blunt mechanical injury from lightning strike, muscle contraction or complication of a fall afterwards.

The clinical manifestations of lightning injuries range from mild burns to severe multiorgan dysfunction and death. We present a case of a young female who was knocked unconscious after a near lightning strike and who sustained subsequent speech impediment, loss of consciousness, muscle spasms and paresthesias.

### Case report

A 28-year-old female presented to the Emergency Department via EMS at 4 p.m. in the afternoon with the chief complaint of a near lightning strike. The patient was outdoors at a field playing Frisbee when lightning struck a nearby tree that was very close to the patient. As per witnesses the patient was thrown back with positive loss of consciousness. When she awoke she complained of a heavy tongue, speech difficulty, difficulty swallowing, tinnitus and diffuse muscle cramps with paresthesias. The patient had been previously healthy with no past medical history and was on no current medications.

On examination the patient was normotensive (116/81 mmHg), tachycardic (111 beats per minute) and tachypneic (28 breaths per minute). Physical exam revealed a well-nourished female, actively crying and upset. The only abnormal finding on physical exam was her speech. It appeared and sounded as though she had congenital deafness. Initial laboratory tests showed total creatine kinase (CK) of 253 units/L, creatinine of 1.07 mg/dL and remaining laboratory tests within normal limits. The electrocardiogram showed a normal sinus rhythm at 82 with normal intervals and no acute ST abnormality. Computed tomography of the head showed no acute injury. The patient received intravenous fluids and intravenous lorazepam for her muscle spasms. Four hours later repeat laboratory tests showed a CK total of 506 U/L and a CK-MB of 7.9 U/L, and creatinine of 0.87 mg/dL. Her speech gradually improved but she continued to complain of severe muscle spasms of her upper and lower extremities. She was admitted overnight to the general medicine service for serial creatine kinase measurements to monitor for rhabdomyolysis as well as for treatment of her myalgias and muscle spasms. Frequent neurological checks would also be implemented.

In the hospital, the patient was monitored on the telemetry floor and no arrhythmias were noted. The total creatine kinase rose to 614 U/L and her creatinine returned to normal after 2 L of intravenous fluids. She continued to have some muscle spasms, which improved with valium. The patient was tolerating oral fluids and ambulated without difficulty after 1 day so she was discharged home to follow-up with her primary care physician. The patient eventually returned back to work and her speech returned to normal. However, she continued to experience neck and shoulder muscle spasms and mild short term memory loss. At 1 year follow-up, she voiced no complaints.

## Discussion

Environmental electrical injuries are relatively common, usually accidental and can generally be prevented. Lightning injuries are a small subset of electrical injuries and are responsible for an average of 300 injuries and 100 deaths per year in the US. Lightning-related fatalities and hospitalizations are underestimated because much of the data is taken from newspaper accounts and many survivors do not seek medical attention. Lightning injuries can be classified as mild, moderate or severe. Mild injuries consist of loss of consciousness, amnesia, confusion and tingling. Moderate injuries may consist of seizures, respiratory arrest and superficial burns. Severe injuries may consist of cardiopulmonary arrest [3].

We presented a case that appeared to involve both the hypoglossal, hypopharyngeal and vagus nerves affecting normal speech pattern after a near lightning strike. The effects on the patient's speech were transient in nature. The remaining effects on the patient's organ systems only seemingly involved muscle spasm, which also cleared as the patient was able to ambulate within 24 h of injury.

The only documented case involving a similar presentation was cited by Baskerkville and McAninch in which a young female was changing an overhead light bulb in an 120 volt light fixture, which led to a low voltage shock and an associated loss of consciousness, and associated lingular deviation and slurred speech [4]. This individual required treatment with carbamazepine and Botox in order to resolve symptoms over time.

These two cases, although different in current of electricity, seemingly had similar pathways affected and ultimately had complete resolution of their symptoms. It appears that in addition to the fatalities associated with electrical injury, near lightning strikes and low voltage electricity can lead to transient peripheral nerve dysfunction. Similarly to the effects on the conduction system of the heart, peripheral nerves can be short circuited, thereby leading to associated dysfunction.

## Conclusion

Lightning strikes are primarily a neurologic injury that affects all three components of the nervous system: peripheral, autonomic and central. Healthcare providers need to be aware of the injury patterns that can occur with lightning strikes in order to provide the best possible care. The patient presented in this case transiently had loss of function of peripheral nerves controlling tongue movement, but ultimately regained the ability to produce speech without further sequelae.

## Consent

Written informed consent was obtained from the patient for publication of this case report and any accompanying images. A copy of the written consent is available for review by the Editor-in-Chief of this journal.

## Competing interests

The authors declare that they have no competing interests.

## Authors' contributions

RF completed the initial case report, BD edited, revised the initial report and added the discussion
